# The bacterial and fungal communities of the larval midgut of *Spodoptera frugiperda* (Lepidoptera: Noctuidae) varied by feeding on two cruciferous vegetables

**DOI:** 10.1038/s41598-022-17278-w

**Published:** 2022-07-29

**Authors:** Li Yuning, Liu Luyang, Cai Xueming, Yang Xianmei, Lin Jintian, Shu Benshui

**Affiliations:** Guangzhou City Key Laboratory of Subtropical Fruit Trees Outbreak Control, Institute for Management of Invasive Alien Species, Zhongkai University of Agriculture and Engineering, 313 Yingdong Teaching Building, Guangzhou, 510225 People’s Republic of China

**Keywords:** Ecology, Microbiology, Molecular biology, Zoology

## Abstract

*Spodoptera frugiperda* is a highly polyphagous pest worldwide with a wide host range that causes serious losses to many economically important crops. Recently, insect-microbe associations have become a hot spot in current entomology research, and the midgut microbiome of *S. frugiperda* has been investigated, while the effects of cruciferous vegetables remain unknown. In this study, the growth of *S. frugiperda* larvae fed on an artificial diet, *Brassica campestris* and *Brassica oleracea* for 7 days was analyzed. Besides, the microbial community and functional prediction analyses of the larval midguts of *S. frugiperda* fed with different diets were performed by high-throughput sequencing. Our results showed that *B. oleracea* inhibited the growth of *S. frugiperda* larvae. The larval midgut microbial community composition and structure were significantly affected by different diets. Linear discriminant analysis effect size (LEfSe) suggested 20 bacterial genera and 2 fungal genera contributed to different gut microbial community structures. The functional classification of the midgut microbiome analyzed by PICRUSt and FUNGuild showed that the most COG function categories of midgut bacterial function were changed by *B. oleracea*, while the guilds of fungal function were altered by *B. campestris* significantly. These results showed that the diversity and structure of the *S. frugiperda* midgut microbial community were affected by cruciferous vegetable feeding. Our study provided a preliminary understanding of the role of midgut microbes in *S. frugiperda* larvae in response to cruciferous vegetables.

## Introduction

Microorganisms are an important part of insects and are colonized on the exoskeleton, in the gut, hemocoel and other tissues^1^. During the long-term evolutionary process, the interdependent symbiotic relationship between insects and microorganisms has been formed^2,3^. In this relationship, insects provide a relatively stable environment and essential nutrition for the microorganisms, while the microorganisms return benefits to the insects in different forms^4^. For example, microorganisms digest the foods ingested by insect hosts and produce the nutrients, including amino acids, vitamins, and nitrogen, for the host’s absorption^5,6^. Some of them protect their insect hosts against various adverse threats, such as pathogen infection and parasitic wasp infestation^7–9^. Increasing reports have evidenced that symbiotic microorganisms also play important roles in the detoxification and metabolism of plant allelochemicals and xenobiotics, such as insecticides and so on^10,11^. Recently, the contribution of symbiotic microorganisms to the reproduction, growth, and waste conversion of the primary insects reared as food and feed, such as black soldier flies, *Hermetia illucens* (Diptera: Stratiomyidae), mealworms, *Tenebrio molitor* (Coleoptera: Tenebrionidae), and crickets, *Acheta domesticus* (Orthoptera: Grylloidea), has also received widespread attention^12^.

Insect gut microbiota attract widespread concern because of their role in contributing to host life-traits^13^. A variety of factors, including host phylogeny, environment, and diet, may influence the gut microbial community and structure^4^. Diet is the most important factor that can rapidly and significantly alter the relationship between the insect host and gut microbiota, as well as have short- and long-term effects on the gut microbial community, taxonomic, and functional associations, demonstrating gut microbiota plasticity^14,15^. Plasticity is apparent in polyphagous insect herbivores. Herbivorous insects have evolved multiple strategies to adapt and degrade to the unfavorable compounds in different hosts, such as the association with symbiont microbiota, which could be useful for exploiting new food sources^4,16^.

The fall armyworm (FAW), *Spodoptera frugiperda* (J. E. Smith), is the most economically important agricultural pest native to tropical and subtropical regions of the Americas^17^. FAW has become a worldwide pest and has been introduced into Africa, Asia, and Oceania in the past few years^18,19^. The larvae are highly polyphagous with a wide host range and could feed on more than 353 plant species^20^. Maize, wheat, rice, sorghum, cotton, and other economically important crops were damaged by the larvae, causing great economic losses and threatening food security worldwide^20^. With advances in high-throughput sequencing technologies, studies of the gut microbiome of *S. frugiperda* associated with different plant hosts have increased in recent years. For example, the effects of soybean and maize on *S. frugiperda* larval midgut bacterial communities have been analyzed, and a more diverse bacterial community was observed when the larvae feed on soybean^21^. The influences of different genotypes of maize (*Zea mays*), including B73, Tx601, and Mp708, on *S. frugiperda* larval midgut community structure and composition were also explored^22^. Significant differences in gut microbial community structure and diversity were exhibited when the larvae fed on different hosts^23,24^. These studies could contribute to the research of the host adaptation of *S. frugiperda* and the development of efficient and environmentally friendly control strategies.

In general, highly polyphagous lepidopteran species have a partially overlapping host range^14^. *Brassica* vegetables are the host plants for the common cutworm, *Spodoptera litura*, a relative of *S. frugiperda*^25,26^. Recently, the biology and biometric characteristics of *S. frugiperda* reared on *Brassica oleracea* var. botrytis (cauliflower) were investigated^27^. Therefore, we speculate that *Brassica* plants may also be the hosts of *S. frugiperda*. To further learn more about the relationships among *S. frugiperda*, *Brassica* plants and microbes, two cruciferous vegetables, including pakchoi (*Brassica campestris* L.) and purple cabbage (*Brassica oleracea* L.), which are the most popular *Brassica* vegetables in China, were selected for the experiments. The effects of these plants on the growth of *S. frugiperda* larvae were investigated. Besides, the changes in the midgut microbial community, including bacteria and fungi, when feeding on these plants were further determined by high-throughput sequencing. Our study provides more basic information for enriching the relationship between the gut microbial community of *S. frugiperda* and plant host fitness. These results could be beneficial to the biology and ecology research of *S. frugiperda*.

## Results

### The growth of *S. frugiperda* larvae on different diets

**Figure 1:**
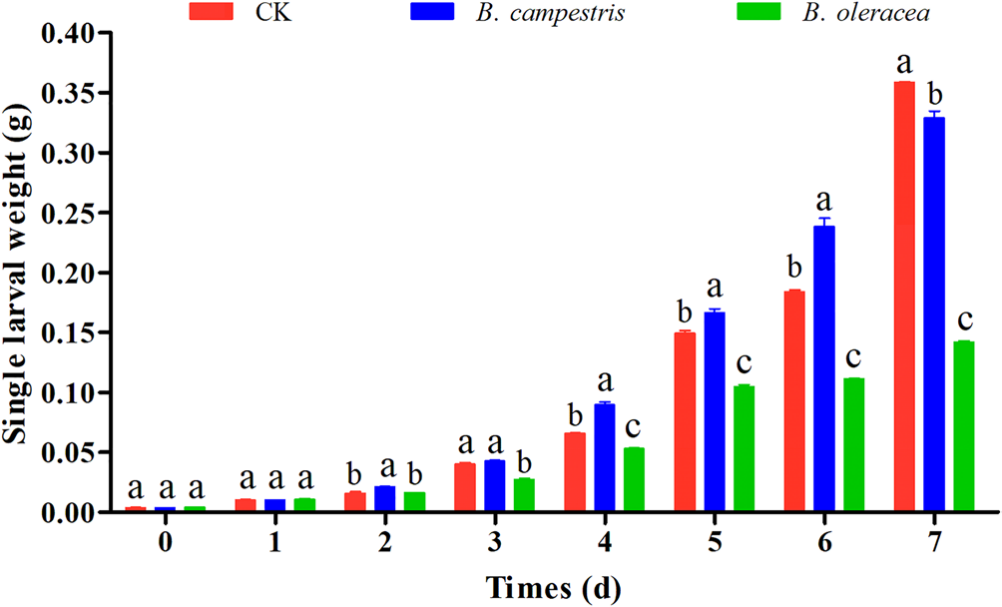
The effects of *B. campestris* and *B. oleracea* on the growth of *S. frugiperda*. The values of single larval weight for every day were shown as mean ± SEM (n = 20). Larvae fed on an artificial diet were used as a control. One-way ANOVA and the DMRT test (*P* < 0.05) were carried out for statistical analysis. Different letters above the bars represent groups with significant differences. The figure was generated with GraphPad Prism software (version 9.0) (https://www.graphpad.com/scientific-software/prism/).

To examine the effects of cruciferous vegetables on the growth of *S. frugiperda* larvae, third instar larvae fed on different diets were weighed daily until day 7. Compared to the control group, the single larval weight in the group fed on *B. campestris* increased significantly in most instances. The average single larval weight decreased significantly after 2 days in the group fed on *B. oleracea*, and the growth inhibitory effect continued until day 7 (Fig. 1). These results indicate that the adaptation of *S. frugiperda* larvae to these two cruciferous vegetables was different.

### Statistics on data sequencing

**Figure 2:**
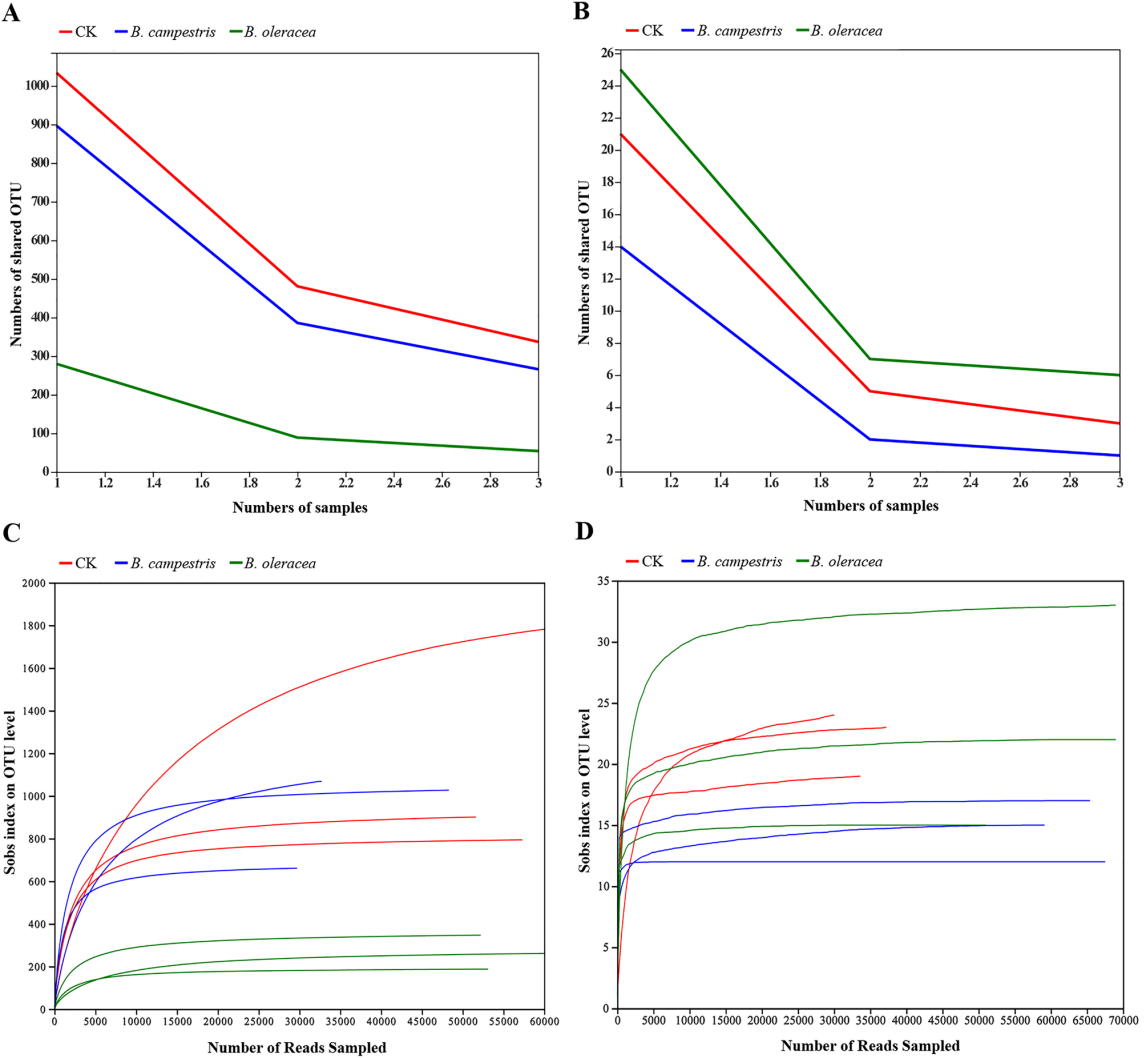
The quality analysis of OTUs in different samples. (**A**) OTU core analysis based on 16S rDNA sequencing. (**B**) OTU core analysis based on ITS sequencing. (**C**) OTU rarefaction curve derived from 16S rDNA sequencing. (**D**) OTU rarefaction curve derived from ITS sequencing. The figure was created by R project Vegan package (version 2.4–3) (https://github.com/vegandevs/vegan/releases/tag/v2.4-3).

**Figure 3:**
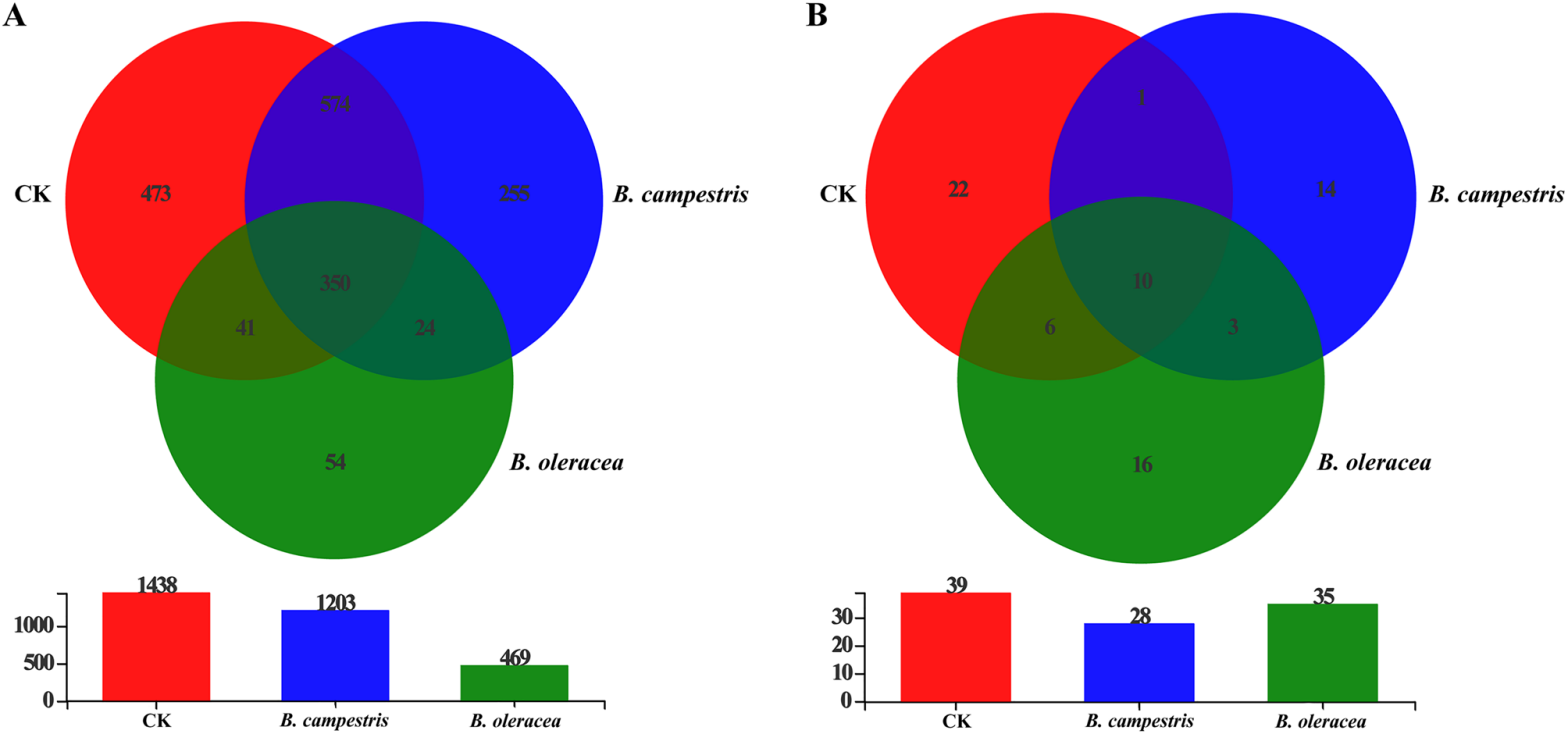
The analysis of the number of microbial species in different groups. (**A**) A Venn diagram and histogram of the bacterial species in different groups. (**B**) A Venn diagram and histogram of the fungal species in different groups. Red rings represent the species from the control group, blue rings represent the species from the group fed on *B. campestris*, and green rings represent the species from the group fed on *B. oleracea,* respectively. The figure was created by jvenn (version 1.0) (http://www.bioinformatics.com.cn/static/others/jvenn/example.html.).

After sequencing, a total of 465,257 and 482,783 high quality 16S rDNA and ITS sequences were obtained (Supplemental Table 1 and 2). The 16S rDNA sequences were classified into 3065 OTUs, 1771 species, 973 genera, 507 families, 312 orders, 144 classes, and 46 phyla. Likewise, all ITS sequences were classified into 117 OTUs, 91 species, 72 genera, 64 families, 44 orders, 22 classes, and 6 phyla. The rarefaction curves and core analysis of these sequences tend to be flat, indicating a sufficient sample number for the sequencing (Fig. 2). Besides, the number of bacterial species in the control group, the group fed on *B. campestris* and *B. oleracea* was 1438, 1203, and 469, respectively. Among them, 473, 255, and 54 species were the unique species in these groups, respectively. A total of 39, 28, and 35 fungal species were found in the control group, the group fed on *B. campestris* and *B. oleracea*, and the number of unique species was 22, 14, and 16, respectively (Fig. 3).

### The microbial composition in the larval midgut

**Figure 4:**
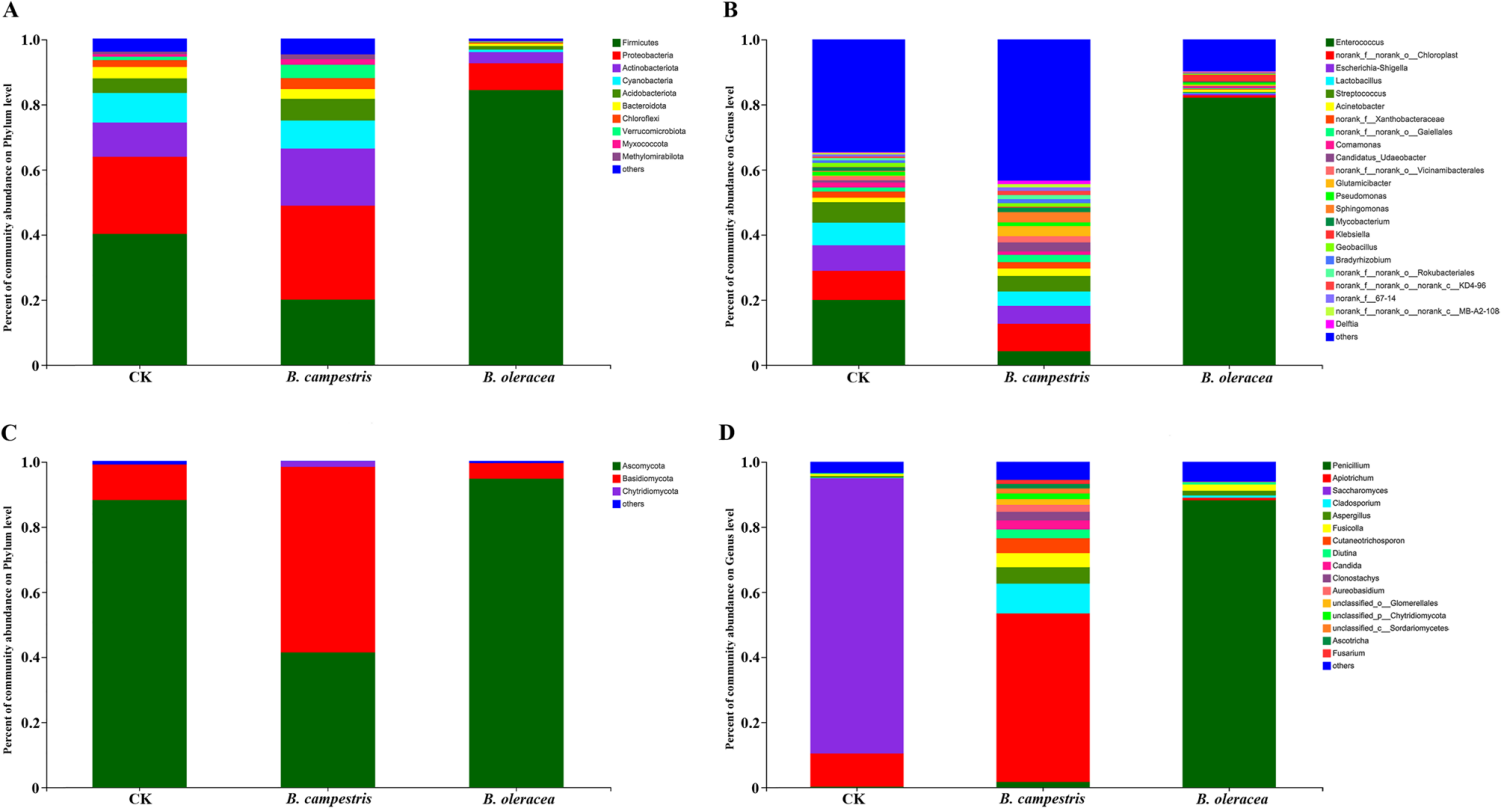
The microbial composition analysis in different groups of *S. frugiperda* larvae. (**A**) and (**B**) represent the bacterial composition in different groups at the phylum and genus levels. (**C**) and (**D**) represent the fungal composition in different groups at the phylum and genus levels. CK: the group fed on an artificial diet, *B. campestris:* the group fed on *B. campestris; B. oleracea:* the group fed on *B. oleracea.* The figure was created by R project Vegan package (version 2.4–3) (https://github.com/vegandevs/vegan/releases/tag/v2.4-3).

In this study, the microbial composition of the larval midgut in different groups was analyzed. For the bacterial composition, Firmicutes, Proteobacteria, Actinobacteriota, Cyanobacteria, Acidobacteriota, Bacteroidota, Chloroflexi, Verrucomicrobiota, Myxococcota, and Methylomirabilota were the main phyla in the larval midgut. Compared to the control group, the abundance of Proteobacteria, Actinobacteriota, Acidobacteriota, Chloroflexi, Verrucomicrobiota, Myxococcota, and Methylomirabilota was increased in the group fed on *B. campestris*, while firmicutes had a decreased abundance. Besides, the bacterial composition in the larval midgut of the group fed on *B. oleracea* was different from that in the control group. Among the main phyla, the abundance of Firmicutes increased significantly, while the others decreased (Fig. 4A). In addition, *Enterococcus*, *Escherichia Shigella*, *Lactobacillus*, and *Streptococcus* were the most abundant genera in the control group, which accounted for 20.00%, 7.84%, 6.95%, and 6.29% of midgut bacteria, respectively. The abundance of these genera was decreased in the group fed on *B. campestris* when compared to the control group. In the group fed on *B. oleracea*, the abundance of *Enterococcus* was increased, which made up 82.19% of the midgut bacteria, while others were decreased (Fig. 4B).

For the fungal composition, Ascomycota and Basidiomycota were the two main phyla in the larval midgut. In the control group, Ascomycota and Basidiomycota accounted for 88.11% and 10.86% of the midgut fungi, respectively. Compared to the control group, the abundance of Ascomycota in the group fed on *B. campestris* was decreased to 41.36%, while the abundance of Basidiomycota was increased to 56.91%. Moreover, the abundance of Ascomycota in the group fed on *B. oleracea* was increased to 94.64%, while the abundance of Basidiomycota was decreased to 4.78%. On a genus level, the control group’s larval midgut fungi were primarily composed of *Saccharomyces* (84.57%) and *Apiotrichum* (10.23%), whereas *Apiotrichum* (51.78%) was the most abundant genera in the group fed on *B. campestris*. Other major genera in the group fed on *B. campestris* included *Cladosporium* (9.15%), *Aspergillus* (5.03%), *Cutaneotrichosporon* (4.58%), *Fusicolla* (4.34%), *Candida* (2.77%), and *Diutina* (2.70%) (Fig. 4C). In the group fed on *B. oleracea*, the most abundant genera were *Penicillium*, which accounted for 88.25% of the midgut fungi (Fig. 4D). These results indicate that different host plants altered the gut microbial community composition of *S. frugiperda* larvae.

### Comparative analysis of samples

**Figure 5:**
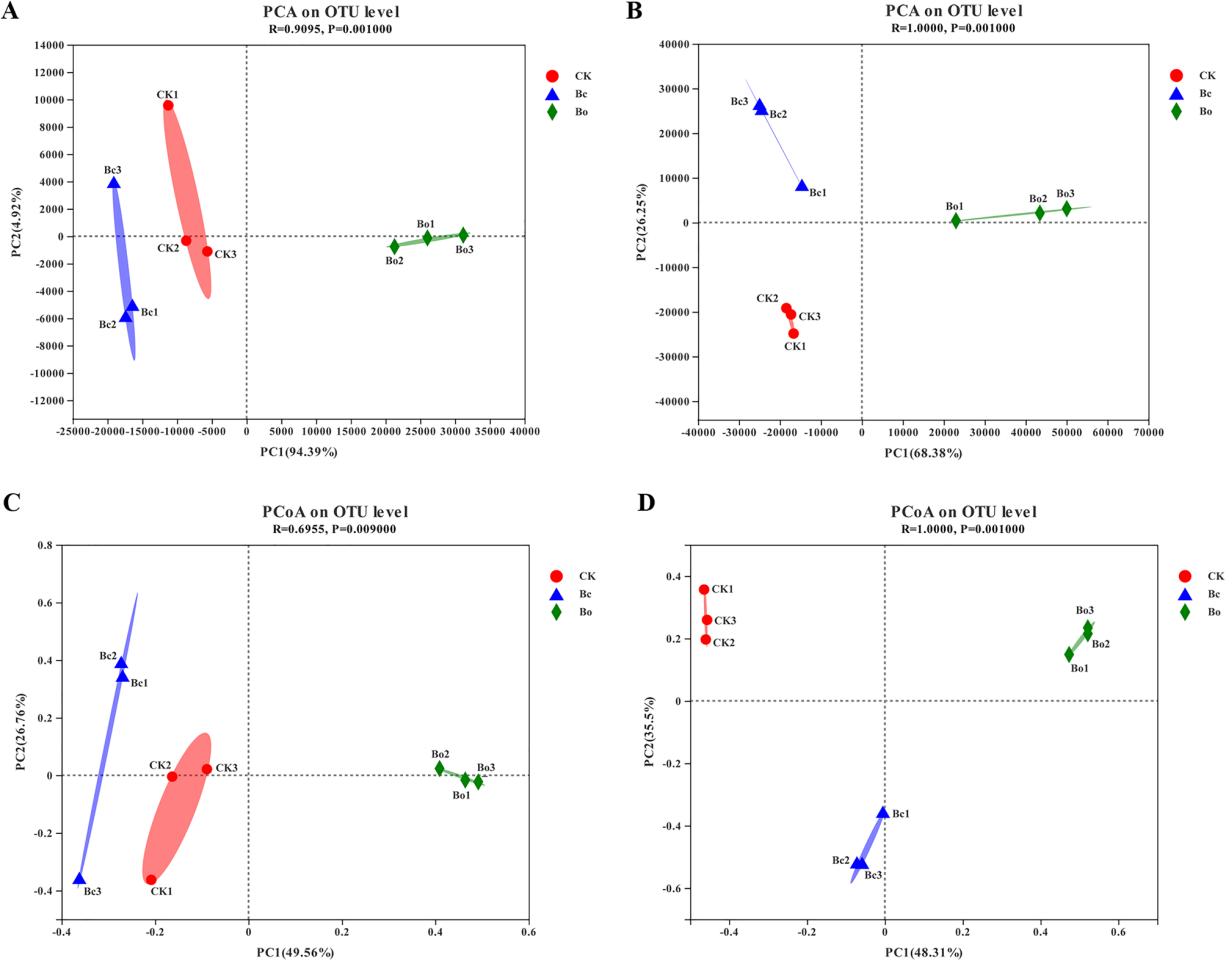
The results of PCA and PCoA analyses of OTUs in all the samples. (**A**) PCA for 16S rDNA data at the OUT level. (**B**) PCoA for 16S rDNA data at the OUT level. (**C**) PCA for ITS data at the OUT level. (**D**) PCoA for ITS data at the OUT level. CK: the group fed on an artificial diet, *Bc*: the group fed on *B. campestris; Bo*: the group fed on *B. oleracea.* The figure was created by R project Vegan package (version 2.4–3) (https://github.com/vegandevs/vegan/releases/tag/v2.4-3).

The *β* diversity analysis of samples in this study was analyzed by Principal Component Analysis (PCA) and Principal Co-ordinates Analysis (PCoA). As shown in Fig. 5A and B, the PCA analysis on the OUT level showed that the midgut bacteria and fungi displayed separate confidence ellipses. Similar results were also observed in the PCoA, where the samples in the same group were clustered together, and the degree of dispersion between different treatment groups was high (Fig. 5C and D). ANOSIM analyses (analysis of similarities) found that the *P*-values in the bacteria and fungi community analysis were 0.009 and 0.001 at the phylum level, respectively. All these results indicate significant differences in microbial community structures among different groups.

### Differential species analysis

**Figure 6:**
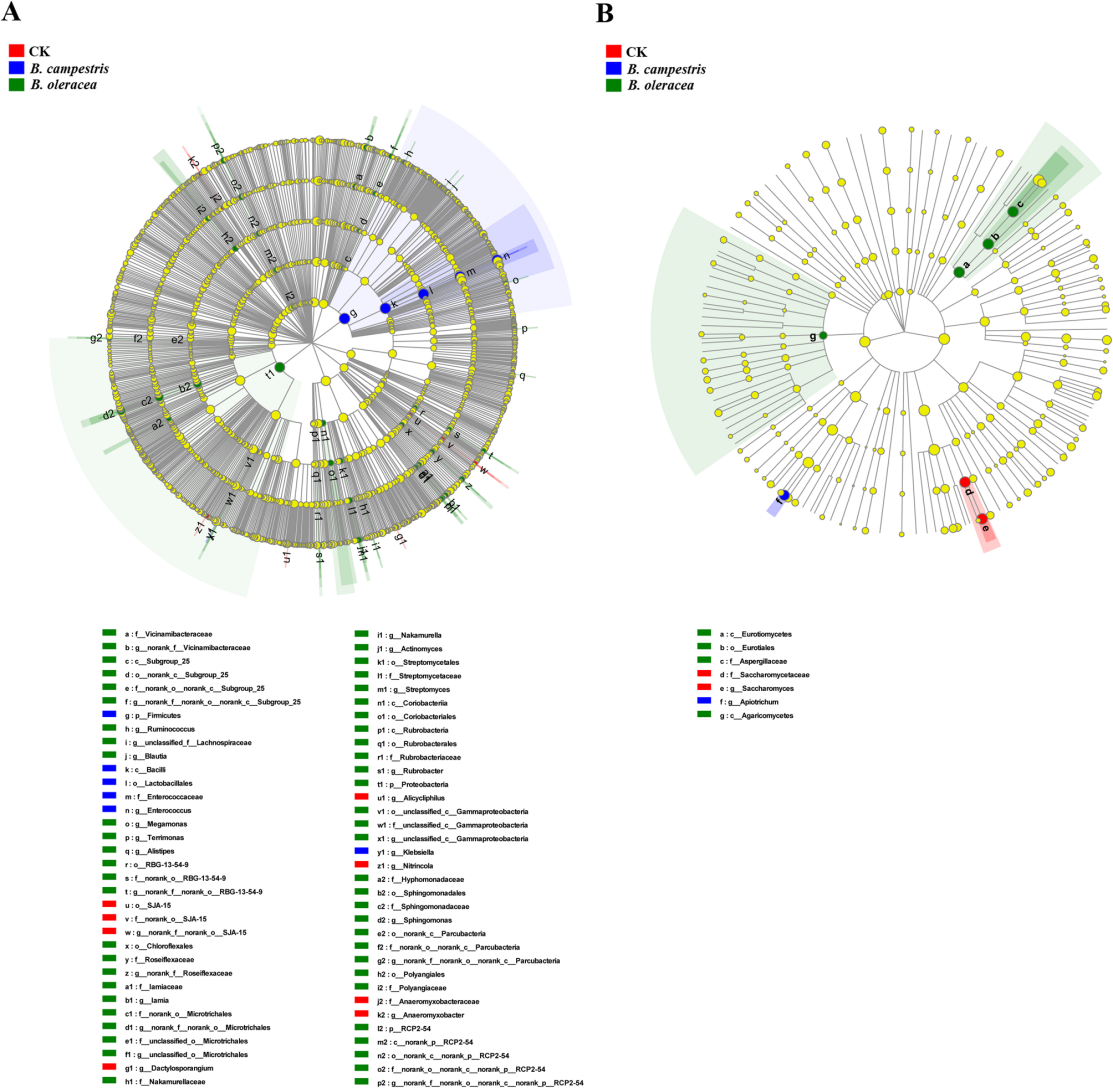
The results of the linear discriminant analysis effect size (LEfSe) cladogram from the phylum to genus. The threshold of Linear Discriminant Analysis (LDA) was set at 2. (**A**) The 16S database’s LEfSe cladogram; (**B**) The ITS database’s LEfSe cladogram. CK: the group fed on an artificial diet, *Bc:* the group fed on *B. campestris; Bo:* the group fed on *B. oleracea.* The figure was created by LEfSe software (version 1.0) (http://huttenhower.sph.harvard.edu/galaxy/root?tool_id=lefse_upload).

Significant differences in bacterial and fungal taxa were also identified by linear discriminant analysis effect size (LEfSe) analyses. The LEfSe cladogram showed that 20 genera contributed to the different midgut bacterial communities in different groups. The genera *Anaeromyxobacter*, *Alicycliphilus*, *Dactylosporangium,* and *Nitrincola* were more abundant in the control group. Similarly, we found an enrichment of *Sphingomonas*, *Nakamurella*, *Ruminococcus*, *Blautia*, *Gammaproteobacteria*, *Actinomyces*, *Streptomyces*, *Microtrichales*, *Alistipes*, *Megamonas*, *Iamia*, *Rubrobacter*, *Terrimonas*, and *Lachnospiraceae* in the group fed on *B. campestris*, as well as *Enterococcus* and *Klebsiella* in the group fed on *B. oleracea* (Fig. 6A)*.* The LEfSe analyses in the midgut fungi community revealed a significant enrichment of *Saccharomyces* in the *B. campestris* group and *Apiotrichum* in the *B. oleracea* group (Fig. 6B)*.*

### Midgut microbial function classification

**Figure 7:**
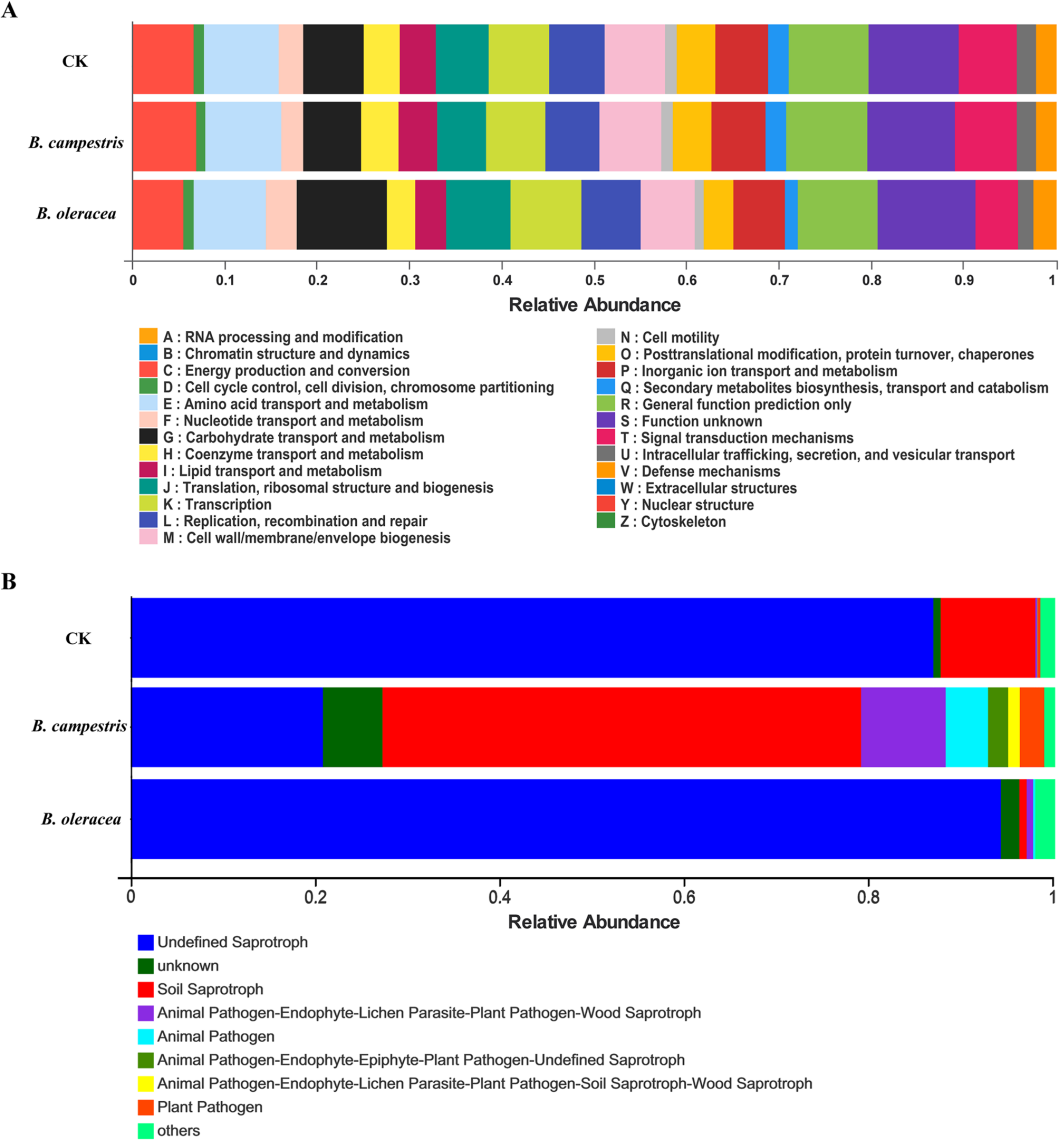
Functional classification of the gut microbial community in *S. frugiperda* larvae*.* (**A**) Bacterial function classification in *S. frugiperda* larval midgut with different diets by the PICRUSt tool. (**B**) Fungal function classification in *S. frugiperda* larval midgut with different diets by the FUNGuild tool. CK: the group fed on an artificial diet, *B. campestris:* the group fed on *B. campestris; B. oleracea:* the group fed on *B. oleracea.* The figure was created by PICRUSt (version 2.2.0) (https://github.com/picrust/picrust2/) and Tax4Fun software (version 0.3.1) (http://tax4fun.gobics.de/).

The tool PICRUSt was used for midgut bacterial function classification, and 25 bacterial function categories were classified. Compared to the control group, no COG category showed a significant difference in the group fed on *B. campestris*, while 22 categories had significantly different abundances in the group fed on *B. oleracea* (Fig. 7A). Among them, the categories of B (Chromatin structure and dynamics), C (Energy production and conversion), D (Cell cycle control, cell division, chromosome partitioning), E (Amino acid transport and metabolism), F (Nucleotide transport and metabolism), H (Coenzyme transport and metabolism), J (Translation, ribosomal structure and biogenesis), L (Replication, recombination and repair), M (Cell wall/membrane/envelope biogenesis), N (Cell motility), O (Posttranslational modification, protein turnover, chaperones), P (Inorganic ion transport and metabolism), Q (Secondary metabolites biosynthesis, transport and catabolism), R (General function prediction only), S (Function unknown), T (Signal transduction mechanisms), U (Intracellular trafficking, secretion) had the *P* value less than 0.01 between control and the group fed on *B. oleracea*, while A (RNA processing and modification) G (Carbohydrate transport and metabolism) I (Lipid transport and metabolism) K (Transcription) had the *P* value less than 0.05. These results indicate that the convergence of midgut bacteria function between control and the group fed on *B. campestris*, while a significant effect on the function of midgut bacteria was observed in the group fed on *B. oleracea*.

For fungal function classification, eight fungal functional guilds were enriched with an abundance of more than 0.01 in samples by the FUNGuild tool. Compared to the control group, the functional guilds of animal pathogen, animal pathogen-endophyte-lichen parasite-plant pathogen-wood saprotroph, plant pathogen, and undefined saprotroph maintained significantly higher abundance in the group fed on *B. oleracea.* The fungal function in the group fed on *B. campestris* was more affected. The guilds of animal pathogen, animal pathogen-endophyte-epiphyte-plant pathogen-undefined saprotroph, animal pathogen-endophyte-lichen parasite-plant pathogen-soil saprotroph-wood saprotroph, animal pathogen-endophyte-lichen parasite-plant pathogen-wood saprotroph, plant pathogen*,* soil saprotroph, and unknown showed different abundance significantly in the group fed on *B. campestris* (Fig. 7B).

## Discussion

With the continuous expansion of the distribution of *S. frugiperda* around the world, the adaptability of larvae to a wide range of hosts has received widespread attention. As a polyphagous pest, a broad host range is essential for its survival and spread^28^. However, different host plants with varying palatability, nutritional content, and presence of secondary metabolites could affect the growth, development, and overall population fitness of *S. frugiperda*^29^. Recently, the adaptation of *S. frugiperda* larvae to many grain and oil crops such as maize, *japonica* or *indica* rice cultivars, sorghum, wheat, cotton, soybean, oilseed rape, and sunflower has been reported^29–32^. Besides, the biological parameters of *S. frugiperda* on other crops, such as three green manure crops, *Astragalus sinicus* L., *Vicia villosa* Roth, and *Vicia sativa* L., and three solanaceous vegetables, *Capsicum annuum* L., *Solanum lycopersicum* Mill., and *Solanum melongena* L., were also investigated^33,34^. In this study, two cruciferous vegetables, *B. campestris* and *B. oleracea,* were selected for feeding the larvae, and the weight changes during the 7 days of feeding were recorded. Our results showed that the larvae have good adaptability on *B. campestris*, but their growth and development are significantly inhibited on *B. oleracea.* To the best of our knowledge, this is the first study to evaluate the effect of cruciferous vegetables (*B. campestris* and *B. oleracea*) on *S. frugiperda.* The significant difference in larvae fed on different plants could be explained by the different nutritional content and presence of secondary metabolites in these two vegetables^20,35^. Given that these two cruciferous vegetables, *B. campestris* and *B. oleracea,* are currently the most popular and consumed vegetables, our results also provide a warning for vegetable cultivation because both types of vegetables can be harmed by *S. frugiperda.*

Microbes are the facultative and/or obligate symbionts for polyphagous insects to overcome the challenges of feeding on different host plants and have beneficial and fundamentally important impacts on insect biology, such as the growth and development, communication, adaption to the environment, and evolution of insects^15,16,36^. In this study, the midgut bacterial and fungal communities of *S. frugiperda* larvae fed on two cruciferous vegetables, *B. campestris* and *B. oleracea*, and an artificial diet, were analyzed. There was a significant difference in the diversity and abundance of the midgut bacterial and fungal communities when *S. frugiperda* larvae fed on different plants, which was similar to the results reported in many previous findings in Lepidoptera^4,23,37^. This is the first time we investigated the effects of cruciferous vegetables on the microorganisms in *S. frugiperda* larvae, which enriched the diversity of the midgut microbial community of *S. frugiperda* and provided a preliminary understanding of the role of midgut microbes in *S. frugiperda* larvae feeding on cruciferous vegetables.

Besides, the composition and diversity of the midgut microbes in *S. frugiperda* larvae with different diets were analyzed. As a food rich in nutrients, the bacterial species in the control group fed on the artificial diet are the most abundant. The number of bacterial species in the group fed on *B. campestris* was close to that in the control group, while it was the lowest in the group fed on *B. oleracea*. Besides, the bacterial diversity of different groups was significantly changed. Many endemic bacterial species were found in different groups. Because of the role of gut microbiota in insect metabolism and growth, the significantly different levels of the diversity of midgut bacteria among these groups could be explained by three reasons: bacteria in diets alter the larval midgut bacterial flora; the nutrients provided by *B. campestris* and *B. oleracea* are not sufficient for the survival of many bacteria in the control group; and the secondary metabolites in *B. campestris* and *B. oleracea* could inhibit the survival of bacteria in the control group, which in turn affects the contribution of bacteria to insects and regulates the growth of insects^29^. Furthermore, there was little difference in the number of fungal species in different groups, while the diversity was significantly plant-associated. The species endemic to different treatment groups may also be derived from the diets. The further function of these endemic species needs to be further explored. Our results further support the conclusion that gut fungal communities could also be affected by the host species^38,39^. Therefore, a revelation emerged that regulating the gut microbiota could be a potential approach for the control of *S. frugiperda*^40^.

The present studies showed that Firmicutes, Proteobacteria, and Actinomycetes are the dominant phyla in the gut bacterial community of *S. frugiperda*, which was similar to that of many lepidopteran insects, including *Lymantria dispar asiatica*, *Lymantria xylina*, *Grapholita molesta*, *Spodoptera exigua*, and so on^2,3,40,41^. The difference in the abundance of these phyla in *S. frugiperda* gut was also observed, which could be explained by different sampling areas, environments, and sequencing technology^42,43^. These results further confirm the indispensability of these bacteria in host insects. According to our results, the similarity in bacteria composition and growth trend observed in the control group and the group fed on *B. campestris* indicates a similar gut microbial environment and function, which further confirms the importance of the microbial environment for growth and development of host insects. While the abundance of Firmicutes, Proteobacteria, and Actinomycetes was changed significantly in the group fed on *B. oleracea* when compared to the control group. In addition, the bacterial composition of the *S. frugiperda* larval midgut at genus level was also changed by different plants. Among them, *Enterococcus*, the common dominant genus in most lepidopteran insects, showed the most abundance in the group feeding on *B. oleracea* when compared to the other two groups. The genus *Lactobacillus* has decreased in abundance significantly in the group fed on *B. oleracea* compared to the other two groups. Previous studies have shown that the functions of these phyla and genera, such as nutrient absorption, energy metabolism, the plant’s secondary metabolites degradation, insect immunity regulation, and so on^44–47^. Our results indicate that the changes in the larval midgut bacterial composition of *S. frugiperda* with *B. oleracea* treatment could be related to the growth of this pest. The specific functions of these phyla and genera in the host plant adaptation of *S. frugiperda* need to be further explored.

Previously, gut fungi were frequently overlooked in microbial research, despite their functions, such as nutrient supply, indigestible compound breakdown, and food detoxification, having been discovered^48,49^. In this study, significant differences existed within groups of fungal communities in the midgut of *S. frugiperda* larvae. *Saccharomyces*, *Apiotrichum*, and *Penicillium* were identified with the most abundance in the control group, the group fed on *B. campestris* and *B. oleracea*. *Saccharomyces* are important fungi for insects in terms of nutrient supply and may be involved in insect development^50–53^. Previous studies of *Apiotrichum* species indicated that they could be involved in lipid biosynthesis, and the degradation and detoxification of toxic substances^54,55^. *Penicillium* is well known for its ability to degrade cellulose, hemicellulose, and lignin^56,57^. These results indicate that the major midgut fungal microbes of *S. frugiperda* larvae may be influenced by the constituents of different diets.

Furthermore, midgut bacterial function classification suggested that many functional categories were changed significantly in the group fed on *B. oleracea* compared to the other two groups, while the fungal guilds with significant changes were observed in the group fed on *B. campestris*. Our results confirmed the functional plasticity of the midgut bacterial and fungal microbes of *S. frugiperda* in response to different diets. Further studies are needed to reveal the details of the function of the midgut microbial community in the host plant fitness of *S. frugiperda* larvae.

In comparison to the control group, *B. oleracea* inhibited the growth of *S. frugiperda* larvae, while the group fed on *B. campestris* showed a similar growth trend. The results of Illumina sequencing revealed that the composition, structure, and function of the midgut microbial community in different groups varied. *B. oleracea* as the diet changed the midgut bacterial community and function, while *B. campestris* altered the fungal community and function significantly. Our preliminary results reveal the plasticity of the midgut microbiome in *S. frugiperda* larvae in response to different plants.

## Materials and methods

### Insect rearing

*S. frugiperda* larvae were collected from a corn field in Conghua District, Guangzhou City, Guangdong Province, China (23°55′N, 113°58′E) on June 20, 2019. The laboratory population had been established without pesticide exposure for more than two years. The larvae were maintained with an artificial diet, and the recipe for a 1 kg diet contained 100 g corn flour, 80 g soy flour, 26 g yeast powder, 26 g agar, 8.0 g vitamin C, 2.0 g sorbic acid, 1.0 g choline chloride, 0.2 g inositol, and 0.2 g cholesterol^58^. The adults were fed with 10% honey water. The insects were cultured in an incubator with stable conditions of a 12-h light / dark cycle at 25 ± 1 °C with 75–85% humidity.

### Treatments

Newly hatched larvae feed on the artificial diet as 3rd instar larvae. Then larvae of the same size were selected for the experiments. The leaves of organic pakchoi and purple cabbage without insecticide exposure were purchased from a local agricultural company (Guangzhou, China). The surfaces of all the leaves were washed with water, sterilized with a 75% ethanol solution, and then rinsed in sterilized distilled water. The larvae fed on the leaves of *B. campestris* and *B. oleracea* were set as two experimental groups, respectively. At the same time, larvae fed on an artificial diet were used as a control group. The larvae were housed individually in the *Drosophila* vials (25 mm diameter, 95 mm height, Crystalgen Inc., USA) to prevent cannibalism, and the diet in the vials was changed every day. Twenty larvae were used as one replicate, and three replicates were set for each group. The body weight of larvae in different groups was recorded daily until day 7.

### Sample collection

The larvae fed on different diets for 7 days were used for sample collection. The larvae were sterilized with a 75% ethanol solution, then washed with sterile water three times. The clean bench (AIRTECH, Jiangsu, China) was exposed to UV for more than 30 min. The larvae were then dissected on a sterile clean bench, and the midgut contents were collected in 1.5 mL sterile centrifuge tubes. The samples were then quickly frozen in liquid nitrogen and stored at − 80 °C. Three biological replicates were performed for each treatment, with 15 larval midgut contents collected per replicate.

### DNA isolation and PCR amplification

The genomic DNA for each sample was isolated with an E.Z.N.A.® soil DNA kit (Omega Bio-tek, Norcross, GA, U.S.) according to the manufacturer’s protocol. The quality of genomic DNA was analyzed with a 1% agarose gel electrophoresis, and a NanoDrop2000 (Thermo Fisher Scientific, Waltham, MA, USA) was used for DNA concentration and purity determination. After passing the quality inspection, all the genomic DNA from different samples was used for subsequent experiments. In order to analyze the changes in the gut bacterial and fungal communities induced by cruciferous vegetables, the 16S rDNA V3-V4 variable region and Internal Transcribed Spacer (ITS) ITS1-ITS2 region amplifications were performed, and the same genomic DNA was used as the template. For 16S rDNA amplification, the primers 338F (5'-ACTCCTACGGGAGGCAGCAG-3') and 806R (5'-GGACTACHVGGGTWTCTAAT-3') were used. Besides, the primers ITS1F (5'-CTTGTTCATTTAGGAAGTAA-3') and ITS2R (5'-GCTGCGTTCTTCATCGATGC-3') were used for ITS amplification. The PCR reaction system for 16S rRNA and ITS amplifications was basically identical except for primers and is as follows: 4 μL of 5 × TransStart FastPfu Buffer, 2 μL of 2.5 mM dNTPs, 0.8 μL of 5 μM forward primer, 0.8 μL of 5 μM reverse primer, 0.4 μL of TransStart FastPfu DNA Polymerase, and 10 ng of genomic DNA. The procedure for PCR amplification was executed at 95 °C for 3 min; 27 cycles of 95 °C for 30 s, 55 °C for 30 s, and 72 °C for 30 s; followed by a stable extension at 72 °C for 10 min, and then kept at 4 °C. Three technical replicates were performed per sample, and three biological replicates were carried out for each treatment.

### Illumina sequencing and sequence analysis

The PCR products were detected with a 2% agarose gel electrophoresis and the target bands were purified with the AxyPrep DNA Gel Extraction Kit (Axygen Biosciences, Union City, CA, USA). After being quantified using a Quantus™ Fluorometer (Promega, USA), the purified PCR products were used for library construction with the NEXTflex™ Rapid DNA-Seq Kit (Bioo Scientific, USA) according to the manufacturer’s protocols. Finally, the amplicons from different libraries were sequenced on an Illumina Miseq PE300/NovaSeq PE250 platform (Illumina, San Diego, USA).

After sequencing, the raw data was quality controlled by removing low-quality reads using fastp software (https://github.com/OpenGene/fastp, version 0.20.0). Then the clean reads were spliced using FLASH software (http://www.cbcb.umd.edu/software/flash, version 1.2.7). The software UPARSE (http://drive5.com/uparse/, version 7.1) was used for the operational taxonomic units (OTUs) cluster and chimera removal with the 97% similarity cutoff. For 16S rDNA sequences, the species taxonomy annotation was performed using the RDP classifier (http://rdp.cme.msu.edu/, version 2.2) and the Silva 16S rRNA database (v138), with the alignment threshold set to 70%. For ITS sequences, the UNITE database 8.0 was selected for the alignment. All the bioinformatics analyses were performed using the online platform of Majorbio Cloud Platform (www.majorbio.com).

### Data analysis

Each treatment was replicated three times, and the results were expressed as mean values ± SEM. One-way ANOVA followed by DMRT and a student’s *t* test were conducted during statistical analyses (*P* < 0.05).

### Ethics declarations

This article does not involve any human participants and/or animals, other than the fall armyworm, *S. frugiperda*.

## Supplementary Information

Supplementary Information.

## Data Availability

The data presented in this study are available in the Supplementary Materials. The raw data of larval midgut bacteria and fungi have been uploaded to NCBI SRA database with the numbers of PRJNA843198 (https://www.ncbi.nlm.nih.gov/bioproject/PRJNA843198) and PRJNA843200 (https://www.ncbi.nlm.nih.gov/bioproject/PRJNA843200).
