# Supplementary material for: The bacterial and fungal communities of the larval midgut of Spodoptera frugiperda (Lepidoptera: Noctuidae) varied by feeding on two cruciferous vegetables
Source: Sci Rep. 2022 Jul 29;12:13063. doi: 10.1038/s41598-022-17278-w (PMC9338029; doi:10.1038/s41598-022-17278-w)
Supplement: Supplementary file 1 — Supplementary Information. [file 41598_2022_17278_MOESM1_ESM.docx]

Supplemental Table 1 Statistical analysis of 16S rDNA sequencing data in different samples

| Sample Name | Seq number | Base number | Mean length | Min length | Max length |
| --- | --- | --- | --- | --- | --- |
| **BC1**  **BC2**  **BC3**  **CK1**  **CK2**  **CK3**  **GL1**  **GL2**  **GL3** | 52,165  31,933  33,990  63,288  55,033  60,315  54,031  53,379  61,123 | 21,832,166  13,336,330  14,229,020  26,675,454  23,159,630  25,408,805  23,099,948  22,765,515  26,104,053 | 418.521346  417.634735  418.623713  421.493079  420.831683  421.268424  427.53138  426.488226  427.074146 | 223  387  277  227  222  277  398  276  219 | 483  441  441  446  470  514  431  432  490 |

Supplemental Table 2 Statistical analysis of ITS sequencing data in different samples

| Sample Name | Seq number | Base number | Mean length | Min length | Max length |
| --- | --- | --- | --- | --- | --- |
| **BC1**  **BC2**  **BC3**  **CK1**  **CK2**  **CK3**  **GL1**  **GL2**  **GL3** | 58,991  67,283  65,137  30,697  37,905  34,182  50,785  68,804  68,999 | 12,589,619  14,147,172  13,505,525  13,442,241  14,581,417  13,649,737  11,069,437  15,015,649  14,845,415 | 213.415928  210.263692  207.340298  437.900805 384.683208  399.325288  217.966663  218.238024 215.15406 | 186  140  146  140  140  140  141  181  144 | 515  480  521  446  481  513  501  521  512 |
